# Scaling up eConsult for access to specialists in primary healthcare across four Canadian provinces: study protocol of a multiple case study

**DOI:** 10.1186/s12961-019-0483-5

**Published:** 2019-09-12

**Authors:** Mylaine Breton, Mélanie Ann Smithman, Clare Liddy, Erin Keely, Gerard Farrell, Alexander Singer, Catherine Lamoureux-Lamarche, Maxine Dumas Pilon, Véronique Nabelsi, Isabelle Gaboury, Marie-Pierre Gagnon, Carolyn Steele Gray, Jay Shaw, Catherine Hudon, Kris Aubrey-Bassler, Justin Gagnon, Élizabeth Côté-Boileau, Paula Louise Bush

**Affiliations:** 1 Centre de recherche Charles-Le Moyne – Saguenay-Lac-Saint-Jean sur les innovations en santé, Université de Sherbrooke, Longueuil Campus, 150 Place Charles-Le Moyne, Office 200, Longueuil, J4K0A8 Canada; 2 C.T. Lamont Primary Health Care Research Center, Bruyère Research Institute, Department of Family Medicine, University of Ottawa, Ottawa, Canada; 3 Department of Medicine, University of Ottawa, Division of Endocrinology/Metabolism, The Ottawa Hospital, University of Ottawa, Ottawa, Canada; 4 Department of Family Medicine, Memorial University, St-John, Canada; 5 Department of Family Medicine, University of Manitoba, Winnipeg, Canada; 6 Family Medicine Center, St-Mary’s Hospital, McGill University, Montréal, Canada; 7 Département des sciences administratives, Université du Québec en Outaouais, Gatineau, Canada; 8 Faculté des sciences infirmières, Université Laval, Québec, Canada; 9 Bridgepoint Collaboratory for Research and Innovation, Lunenfeld-Tanenbaum, Research Institute, Sinai Health System, University of Toronto, Institute of Health Policy, Management and Evaluation, Toronto, Canada; 10 Women’s College Hospital, Institute for Health System Solutions and Virtual Care, Toronto, Canada; 11 Centre de recherche du CHUS, Université de Sherbrooke, Sherbrooke, Canada; 12 Department of Family Medicine, McGill University, Montréal, Canada

**Keywords:** Access to healthcare, eConsult, scaling up, primary care, electronic consultation, process research, implementation, diffusion of innovation, policy

## Abstract

**Background:**

Canada has been referred to as the land of ‘perpetual pilot projects’. Effective innovations often remain small in scale, with limited impact on health systems. Several innovations have been developed in Canada to tackle important challenges such as poor access to services and excessive wait times – one of the most promising innovations that has been piloted is eConsult, which is a model of asynchronous communication that allows primary care providers to electronically consult with specialists regarding their patients’ medical issues. eConsult pilot projects have been shown to reduce wait times for specialist care, prevent unnecessary referrals and reduce health system costs. eConsult has been spread throughout Ontario as well as to certain regions in Manitoba, Quebec, and Newfoundland and Labrador.

Our aim is to understand and support the scale-up process of eConsult in Ontario, Quebec, Manitoba, and Newfoundland and Labrador. Our specific objectives are to (1) describe the main components of eConsult relevant to the scale-up process in each province; (2) understand the eConsult scale-up process in each province and compare across provinces; (3) identify policy issues and strategies to scaling up eConsult in each province; and (4) foster cross-level and cross-jurisdictional learning on scaling up eConsult.

**Methods:**

We will conduct a qualitative multiple case study to investigate the scaling up of eConsult in four Canadian provinces using a grey literature review, key stakeholder interviews (10 interviews/province), non-participant observations, focus groups and deliberative dialogues. We will identify the main components of eConsult to be scaled up using logic models (obj. 1). Scaling up processes will be analysed using strategies adapted from process research (obj. 2). Policy issues and strategies to scale-up eConsult will be analysed thematically (obj. 3). Finally, a symposium will foster pan-Canadian learning on the process of scaling up eConsult (obj. 4).

**Discussion:**

This study will likely increase learning and support evidence-based policy-making across participating provinces and may improve the capacity for a pan-Canadian scale-up of eConsult, including in provinces where eConsult has not yet been implemented. This work is essential to inform how similar innovations can reshape our health systems in the evolving information age.

## Background

Canada has been referred to as the land of “*perpetual pilot projects*” [1], where successfully tested innovations are seldom scaled up to the health system level. Effective innovations often remain small in scale and incapable of improving healthcare and health for a larger population [2, 3]. Meanwhile, health systems struggle to tackle important challenges such as poor access to services, excessive wait times and rising healthcare system costs. Consequently, there is growing research and policy interest in the scaling up of effective innovations [4, 5].

While the terms ‘spread’ and ‘scale-up’ are sometimes used interchangeably, similarly to other authors, we consider ‘spread’ to be more of an organic adoption and replication of an innovation within a health system, whereas we consider ‘scale-up’ to entail deliberate efforts to address the system-level policy and infrastructure issues of a large-scale roll-out of an innovation [6–10]. As such, scaling up an innovation implies an iterative series of decisions, events and actions.

Several recent studies have identified success factors for scaling up promising innovations, including stakeholder engagement, monitoring and evaluation systems, sustainable funding models, leadership, political will, well-defined strategies, supportive infrastructure and policy, sufficient and stable human resources, effective governance, and integration with existing services [5, 8, 11]. A number of conceptual frameworks for scaling up innovations have been developed [5–7, 12, 13]; however, few empirical studies have been conducted in high-income settings like Canada [12]. There is also limited evidence regarding the iterative nature of scale-up processes. Hence, despite the growing interest in scaling up innovations, there remains a poor understanding of how innovations are scaled up in real-world contexts [4, 8, 12, 14]. Failure to move from proven small-scale innovations to innovations scaled up at health system levels has been attributed to this lack of understanding of scale-up processes as well as to limited knowledge exchange across policy and delivery levels, and between jurisdictions [1, 4, 6, 8, 15]. There is, therefore, a need to conduct research to better understand the process of scaling up innovations, while considering real-world contexts and the iterative nature of scale-up processes, and to foster cross-level and cross-jurisdictional exchanges.

### eConsult: a real-world scale-up experiment in Canada

Poor access and excessive wait times for specialist care is one of the most significant challenges of Canadian health systems [15–18]. Poor access to specialists can lead to anxiety, pain, deterioration in health, delays in diagnostics, duplication of services and dissatisfaction of both patients and providers [16, 19]. In a recent Commonwealth Survey, Canada was ranked the worst out of 11 countries for wait time to see a specialist (56% of Canadian patients waited at least 4 weeks). Within Canada, the situation was most dire in Newfoundland and Labrador, Manitoba, Quebec and Ontario with, respectively, 67%, 62%, 59% and 57% of patients waiting at least 4 weeks [20].

One of the most promising innovations that has been piloted in Canada to improve access to specialist care is eConsult [17, 21, 22]. eConsult is a model of asynchronous communication that allows primary care providers (e.g. family physicians, nurse practitioners) to electronically consult with specialists regarding their patients’ medical issues in a wide range of areas, including psychiatry, dermatology, geriatrics, paediatrics, cardiology, oncology, palliative care, sports medicine and clinical pharmacy. eConsult pilot projects have been shown to reduce wait times for specialist care, prevent unnecessary referrals, increase provider and patient satisfaction, and reduce health system costs [15, 16, 21–23].

The model of eConsult first developed and piloted in the Champlain region in Ontario was the Building Access to Specialists through eConsult (BASE) model [17]*.* Given that the pilot projects were shown to be effective, efficient, acceptable and feasible, the spread and scale-up of eConsult has garnered increasing momentum across Canada [24–26]. Between 2016 and 2018, teams in Manitoba, Quebec, Ontario, and Newfoundland and Labrador worked to spread eConsult to new clinics and regions as part of the Connected Medicine Initiative, supported by the Canadian Foundation for Health Improvement, in collaboration with the College of Family Physicians of Canada, the Royal College of Physicians and Surgeons of Canada, and Canada Health Infoway [26]. In addition, three pan-Canadian symposiums have been held with providers, patients, researchers and policy-makers to reflect on the implementation, spread and scale-up of eConsult across the country [27]. Key issues for scaling up eConsult identified during these symposiums include provider remuneration, patient privacy, delivery of care and interjurisdictional medical licensing [27]. Scaling up eConsult has been identified as a priority by provincial governments of Ontario, Quebec, Manitoba and Newfoundland, and Labrador as well as by several professional associations, including the Quebec College of Family Physicians and the Association of Registered Nurses of Newfoundland and Labrador. Hence, it seems that there is a momentum in scaling up eConsult in Canada, with four provinces being actively involved and currently at different phases of the scale-up process (i.e. assessing, developing, preparing and implementing plans to do so). This offers a unique opportunity to conduct embedded research on the process of scaling up an innovation in real-world contexts, while contributing to cross-jurisdictional learning.

### Study objective

The aim of this study is to understand and support the scale-up process of eConsult among four provinces at varying phases of the scale-up process, with a particular focus on provincial-level policy.

Our specific objectives are to:

1. Describe the main components of eConsult relevant to the scale-up process in each province;
2. Understand the eConsult scale-up process in each province and compare processes across provinces;
3. Identify policy issues and strategies to scaling up eConsult in each province;
4. Foster cross-level and cross-jurisdictional learning on scaling up eConsult.

## Methods

### Framework of the scale-up process

**Fig. 1:**
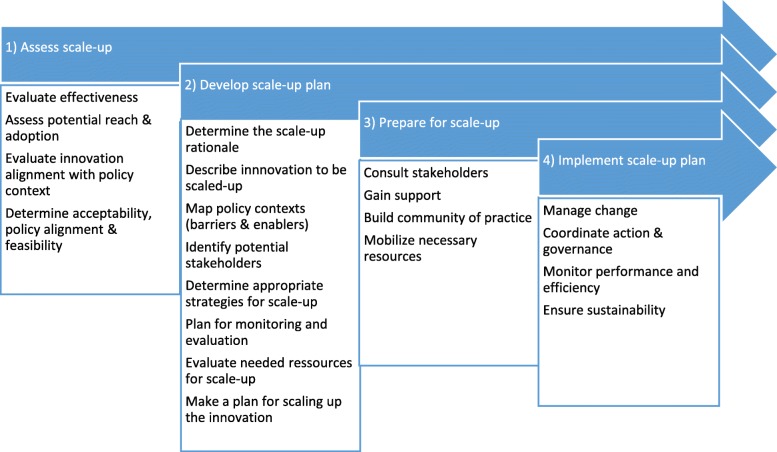
Phases in the scaling-up process (adapted from Milat et al. [12])

To inform our study, we will use a framework developed by Milat et al. [12] that divides the scale-up process into four phases (Fig. 1), as follows: (1) ‘assess scalability’ to determine an innovation’s effectiveness, acceptability and feasibility; (2) ‘develop a scale-up plan’ that can be used to gain support and mobilise resources, (3) ‘prepare for scale-up’ to identify ways of securing resources needed for going to scale and building support for scale-up; and (4) ‘implement the scale-up plan’. Although other frameworks exist [5, 28–30], Milat’s framework is relevant to our study because it was developed specifically to examine “*how scaling-up decision-making and processes occur in high-income countries*”([12], p. 2) and has been adapted for practical use with policy-makers, healthcare providers and other stakeholders.

### Overall study design

This study protocol was written according to the COnsolidated criteria for REporting Qualitative studies (COREQ) checklist [31]. We will conduct a qualitative multiple case study [32] to investigate the contemporary and complex phenomenon of scaling up eConsult within real-world contexts. This design is useful to understand what has happened or what is happening, and why and how it happened, particularly when the boundaries between the phenomenon of interest and the context are blurred [32]. It is therefore well-suited to understand the process of scaling up eConsult in different contexts. We will study the scale-up process in four jurisdictions, namely (1) Ontario, (2) Quebec, (3) Manitoba, and (4) Newfoundland and Labrador. These jurisdictions were selected because they initiated the spread and scale-up of eConsult as part of the Connected Medicine Initiative and sufficient discussions, decisions, events and/or actions pertaining to scale-up have taken place in these provinces to contribute rich data to our study. Variations in the scale-up processes and contexts will allow for interesting comparative analysis and will provide opportunities for cross-jurisdictional learning. The methods for each objective are described in more detail in the following sub-sections. Table 1 summarises our objectives and planned data collection, analysis and products.

**Table 1:** Summary of objectives, data collection, data analysis and products

Objectives	Data collection	Data analysis	Products
1. Describe the main components of eConsult relevant to the scale-up process in each province	Grey literature Semi-structured interviews	Thematic analysis Validation with key stakeholders	Logic model of eConsult for each province (with variations, if any) Matrix comparing key components of eConsult relevant to the scale-up process across provinces
2. Understand the eConsult scale-up process in each province and compare processes across provinces	Grey literature (same as Objective 1) Semi-structured interviews (same as Objective 1) Non-participant observations	Process research approach: narrative summary, visual mapping strategy Validation with key stakeholders Cross-case analysis and validation	Narrative summary for each province Visual map of key events, decisions and actions in the scale-up process for each province Comparative matrix of scale-up phases across provinces
3. Identify policy issues and strategies to scale-up eConsult in each province	Observations, semi-structured interviews (same as Objectives 1 and 2) Focus group	Thematic analysis	Policy briefs with a list of policy issues and strategies to support scale-up of eConsult in each province
4. Foster cross-level and cross-jurisdictional learning on scaling up eConsult	Deliberative dialogue (pan-Canadian symposium)	Cross-case analysis and validation	Summary of cross-jurisdictional learning on scale-up process

#### Objective 1. Describe the main components of eConsult relevant to the scale-up process in each province

As a first step to understanding the scale-up process, we will describe the main components of the eConsult innovation and identify those that are relevant to the provincial scale-up process. For each province, we will build a logic model describing the main components of eConsult (capturing important variations, if any) and will use the logic models in interviews with key stakeholders to identify the key components of eConsult that are relevant and must be addressed in the scale-up process. The logic models will be elaborated using Mitchell and Lewis’ Manual to Guide the Development of Local Evaluation Plans [33]. This particular logic model involves a simple diagram of the main components of interventions and is widely used in research on primary healthcare interventions in Canada, particularly for use with various stakeholders [34, 35].

Given that a substantial amount of work has been done to evaluate the implementation and effectiveness of eConsult pilots, we will first search the grey literature (Canadian Research Index, TRIP database, Des Libris – The Canadian Electronic Library, Google, Google Scholar, and websites of governments, health institutions, research institutes and professional associations), use snowballing and ask knowledge users and key stakeholders for documents describing the main components of eConsult in each province [36]. We will conduct semi-structured interviews with key stakeholders to member-check our description of eConsult and to identify the key components that are relevant to the scale-up process in their province. We will ask the knowledge users and investigators on our team to identify stakeholders with relevant and diverse knowledge and experience of eConsult in each province (e.g. clinical leaders, professional associations, policy-makers, stakeholders involved in technology and infrastructure, patient partners). We will use snowballing to identify additional participants. Data collection will continue until we reach saturation (we anticipate 10 interviews per case).

Based on an iterative mixed inductive and deductive approach, we will conduct thematic analysis of the documents and interviews [37]. Data will be summarised in logic models for each province (capturing variations, if any) and a matrix will be used to compare key components of eConsult identified as relevant to the scale-up process across provinces.

#### Objective 2. Understand the eConsult scale-up process taking place in each province and compare processes across provinces

For each case, we will analyse grey literature documents (see Objective 1) as well as observe key meetings related to eConsult scale-up efforts (e.g. meeting preparation documents, minutes, agendas) (see Table 2 in Appendix 2 for the observation grid). In addition, we will conduct semi-structured interviews with key stakeholders (see Objective 1 for recruitment strategy). The interview guide questions related to Objective 2 will focus on the phases of the scale-up process (see Appendix 1) and will be adapted according to stakeholders’ roles. Patient partners and knowledge users will be involved in the development of the interview guides to ensure the relevance and clarity of questions.

In the first step of analysis, a narrative strategy [38] will be used to reconstruct the chronological ‘story’ for each case. The narrative summaries will be used to capture important contextual elements and their influences on the scale-up process and to provide a detailed description of the different events, decisions and actions of the scale-up process.

In the second step of analysis, to further reduce the data, a visual mapping strategy to represent key events, decisions and actions in the eConsult scale-up process in each jurisdiction will be created. This will provide a visual and chronological representation of various components and strategy. This method is well-suited for working closely with a variety of stakeholders [38]. Drafts of the visual maps will be presented to stakeholders for member-checking during a focus group in each province (see Objective 3) to increase trustworthiness [39].

In the third step of analysis, the visual maps for each of the four cases will be compared and contrasted to look for common and divergent sequences, representations of Milat’s phases of scaling up and patterns of events, decisions and actions. We will use a coding matrix to compare scale-up processes across provinces. Analysis will be performed by two research team members and discussed at team meetings to achieve consensus. The aim of this step will be to develop a more general understanding of the eConsult scale-up process [38]. These findings will be presented to stakeholders at the pan-Canadian deliberative forum (see Objective 4) where they will be asked to member-check the information.

#### Objective 3. Identify policy issues and strategies to support the scale-up of eConsult in each province

As the scaling up of eConsult is an ongoing process, our study will provide an opportunity to support scale-up efforts in each province. We will identify policy issues in scaling up eConsult and strategies to support the scale-up process. Observations and semi-structured interviews (conducted for Objectives 1 and 2) will be used to identify policy issues of scaling up eConsult in each province. These emerging themes, along with the phases in Milat’s conceptual framework, will be used to develop an adapted focus group discussion guide around past and current policy issues and existing or potential strategies to address these issues. Patient partners and knowledge users will be involved in developing discussion guides to ensure relevance and clarity of questions. A focus group of 8 to 10 participants will be organised in each province. Participants will be key stakeholders with various roles and perspectives (e.g. clinical leader, policy-makers, patient partners, etc.) to generate rich discussions around policy issues and strategies [40]. Participants may be the same as stakeholders and interviewed as appropriate. Focus group discussions will last approximately 90 min and will be held in person or by videoconference. Thematic analysis will be conducted [37] using a codebook based on key issues identified in previous reviews [5, 8, 41] and on the phases in Milat’s conceptual framework. We will summarise our findings in policy briefs for each province. Knowledge users and patient partners will be involved in creating these briefs to improve their clarity and relevance to contexts in each province.

#### Objective 4. Foster cross-level and cross-jurisdictional learning on scaling up eConsult

To share the results of the study and to foster cross-jurisdictional knowledge exchange, a 1-day face-to-face pan-Canadian symposium will be organised between researchers, policy-makers, healthcare providers and patient partners. For the past 3 years, annual face-to-face meetings have been organised in Ottawa with a wide-range of stakeholders to present findings and discuss issues surrounding eConsult. In November 2018, over 60 stakeholders from across Canada attended the eConsult symposium, including those from provinces just starting to implement eConsult and from provinces that have not yet implemented it.

We will host a similar national symposium in 2020 in Ottawa. Representatives from national organisations such as the Canadian Medical Association, Primary and Integrated Healthcare Innovation (PIHCI) networks, Canadian Nurses Association, College of Family Physicians of Canada, and Canada Health Infoway will be invited, as well as providers, researchers, patient partners and provincial policy-makers. We expect approximately 50–60 stakeholders to participate. This meeting will be organised in partnership with the Canadian Foundation for Healthcare Improvement, which has expertise and experience in organising pan-Canadian knowledge exchange events and workshops with various key stakeholders. The objectives will be to deepen our understanding of the scale-up process of eConsult across Canada, discuss policy issues and strategies to scale-up eConsult, and foster learning across jurisdictions and health system levels. The experience of provinces further along in the scale-up process may be valuable to other provinces in early phases of the process and may help them prepare for the next phases of scaling up. The meeting will be conducted as a deliberative dialogue [42], which provides a way to involve stakeholders with diverse views in a discussion around issues, to share different experiences and to find promising avenues for actions [43]. The deliberative dialogue will be used to share learning across jurisdictions, identify recommendations for improving current scale-up processes in each jurisdiction, and identify key strategies to facilitate the scale-up of eConsult in different contexts. The symposium will be recorded and summarised in detailed notes. Data will be used to enrich our understanding of the scale-up process (Objective 2), policy issues and strategies to scale up eConsult in each province (Objective 3). In addition, a summary of the key learnings from the symposium – reviewed by knowledge users and patient partners – will be disseminated across Canada.

## Discussion

The aim of this study is to understand and support the scale-up process of eConsult by fostering cross-jurisdictional and cross-health system-level knowledge exchanges between provinces at varying phases of the scale-up process, with a particular focus on provincial-level policy. By engaging patient partners, knowledge users (including stakeholders involved in the pilot projects and in scale-up processes), we will learn from various stakeholders’ experience and knowledge as well as increase the potential for this study to inform and support the scale-up process.

This study will fill an important gap in the literature as it involves a multiple case study examining iterative processes of scaling up an innovation at the health system level in four different contexts. Given the growing interest from policy-makers in scale-up, our research may allow for more evidence-based policy-making and may influence the scale-up process of eConsult in Ontario, Quebec, Manitoba, and Newfoundland and Labrador. In addition, by fostering cross-jurisdictional exchanges, our project will increase learning across provinces and may improve the capacity for the pan-Canadian scale-up of eConsult, including in provinces where eConsult has not yet been implemented. Scaling-up eConsult has the potential to improve access to specialist care across the country.

As eConsult is part of a large range of eHealth-related innovations, this work is essential to inform how similar advances in this emerging field can reshape our health systems in the evolving information age. Many eHealth innovation pilot projects have shown promising results for improving access to care [44], but few have been scaled up at a health system level. Given the rapid pace of technological change, eHealth innovations often face unique policy issues related to scaling up. Our study will help understand how we can move from innovations piloted as part of research to large scale health system changes. Thus, a cross-jurisdictional study on scaling up eConsult at a system level may provide important lessons to inform the scale-up of other evidence-based eHealth technologies.

## Data Availability

Not applicable.

## Appendix 1 – Preliminary semi-structured interview guide

****Explain the aim of the study, risks and benefits. Obtain consent. Clarify what is meant by scale-up of eConsult.****

**Role/perspective**

1. What is your title/position?
2. How have you been involved in eConsult?

**Process of scaling up**

3. Can you tell me the story of eConsult in **province** up to now?
4. Can you describe how eConsult has been or is planning to be scaled up in **province*?*

*General probes to identify key actors, events, decisions, actions* – How did it start? Who were the main leaders? Who was involved? What were the turning points? What was the context? What were the main milestones? Did you have any set-backs? What strategies were used to overcome X?

****Ask questions that apply or that have not been touched upon.****

5. How did the conversation about scaling up eConsult start? How did you decide it was a good innovation to scale up?
  - Probes: effectiveness, potential reach and adoption, alignment with policy and context, acceptability, feasibility
6. What were the steps involved in developing a plan to scale up eConsult?
  - Probes: rationale for scale-up, policy context, stakeholders, appropriate strategies, plan for monitoring and evaluation, resources, plan for scaling up
7. When did **province** actually start preparing to scale up eConsult? How did **province** prepare for scale-up?
  - Probes: stakeholder consultation, community of practice, mobilise resources
8. When did **province** implement the scale-up plan? How did the eConsult scale-up unfold?
  - Probes: change management, governance, monitoring, sustainability
9. Where would you say **province** currently is in scaling up eConsult?

**Design**

10. **Present the draft logic model** Of the components presented here and that you have mentioned, which are essential to the scale-up of eConsult at a provincial level? Which can be adapted to context?
  - Are there regional or local variations in the design of eConsult within your province?
11. Are there components from the pilot projects that needed/will need to be adapted for provincial scale-up? Can you explain why they need/ed to be changed?

**Policy issues**

12. What are the main policy issues of scaling up eConsult in **province*?*
13. What strategies have you used to overcome these issues? What other strategies do you think could help address these issues?
14. What role have patient partners played in scaling up eConsult up to now? How do you think patient partners and the population can support future scale-up efforts?
15. How has research contributed to scaling up eConsult?
16. What are recommendations you would make for the scale-up of eConsult?
  - What issues do you think are not talked about enough in the scale-up of eConsult?
17. What do you think the next step will/should be in scaling up eConsult?
18. Is there anything else I did not ask about, that you think we should know about eConsult?

## Appendix 2

**Table 2:** Non-participant observation grid

Potential issues	Challenges	Solutions/strategies
Remuneration (fees for GPs, fees for all type of providers, payment models, etc.)		
Recruitment of specialists (difficulties to recruit specialists, difficulty to recruit among specific specialties, etc.)		
Information technology (integration into electronic medical records, difficulties with the platform, patient access to the platform, etc.)		
Budget (financing of pilot projects, financing difficulties related to scale-up, government support, etc.)		
Privacy (Concerns regarding patients’ privacy, barrier to use the platform, security of the platform, etc.)		
Delivery of services (Definition of a ‘specialist’, which specialty is available, quality assurance, interjurisdiction eConsults, etc.)		
Provincial governance structure		
Monitoring – Dashboards		
Other issues		
Outcomes’ monitoring/Research		
Milat’s scale-up phases [12]		
Phase 1		
Scalability assessment		
Phase 2		
Develop scale-up plan		
Phase 3		
Prepare for scale-up		
Phase 4		
Implement scale-up plan		
Notes		
